# Mechanical Properties of Perlite Concrete in Context to Its Use in Buildings’ External Walls

**DOI:** 10.3390/ma17235790

**Published:** 2024-11-26

**Authors:** Olga Szlachetka, Justyna Dzięcioł, Marek Dohojda

**Affiliations:** Institute of Civil Engineering, Warsaw University of Life Sciences—SGGW, 166 Nowoursynowska Street, 02-787 Warsaw, Poland; justyna_dzieciol@sggw.edu.pl (J.D.); marek_dohojda@sggw.edu.pl (M.D.)

**Keywords:** expanded perlite, perlite concrete, load-bearing wall, compressive strength, circular economy

## Abstract

Nowadays, much of the attention paid to building construction is focused on sustainability and environmental protection. The materials applied in construction should be safe and free of toxins, but they should also follow the idea of circular construction. Quests for materials with an appropriate structure and composition, unifying features of a construction, insulation (thermally and acoustically), and environmentally friendly material turned our attention in this paper toward expanded perlite (EP). This study aimed to analyze the results of the experimental determination of the basic physical and mechanical parameters of expanded perlite and pure perlite concrete blocks (PPC), i.e., containing 100% EP instead of sand, while in contrast, most existing studies focus only on the partial replacement of sand with EP. This research aims to confirm that PPC containing 100% EP is the product that meets the requirements for load-bearing walls in single-family buildings in European countries such as Poland. The study aimed to determine the procedure for preparing the samples of PPC, i.e., the mixing procedure, the displacement speed during compaction, and the maximum loading force during compaction. It was determined that the appropriate speed of compaction to form the samples is 15 mm per minute, i.e., the same as during the compressive strength tests. The maximum compaction force of 10,000 N during the preparation of samples at a speed of displacement of 15 mm per minute guarantees a compressive strength greater than 3 MPa for dry density class 650, and the method of forming the samples in a single layer, i.e., solid samples.

## 1. Introduction

Energy efficiency is central to modern building practices, driven by growing public awareness and tighter regulations. Policies such as the EU Green Deal aim to achieve carbon neutrality by 2050 by improving building energy efficiency. For example, such regulations in Poland [1] require that the heat transfer coefficient for external walls in single-family homes do not exceed 0.20 W∙m^−2^ K^−1^, starting in 2021. These standards push builders to reduce conventional energy consumption, minimize CO_2_ emissions, and cut other pollutants resulting from fossil fuel combustion. The construction industry has responded with advanced materials and techniques to create highly insulative, energy-efficient building envelopes that retain heat, reducing overall energy use. Contemporary buildings should incorporate historical and modern technologies focusing on ecology and environmental protection. Eco-friendly building practices now emphasize reducing environmental impact at every construction stage—from material production to post-demolition recycling. Building materials are carefully selected for health and sustainability, favoring natural (clay, stone, straw) and recycled options.

There is increasing talk of natural building, green building, and bio-building. Bio-building, emerging in the 1970s in Europe, promotes a harmonious relationship between humans, buildings, and nature by using materials and techniques that are healthier and environmentally friendly. Minimizing the harmful effects of civilization on the environment and using renewable energy sources are the courses of action and the central tenets of so-called green buildings (also often referred to as green, sustainable, or integrated), which are intended to be friendly to people and the environment. Certification systems like BREEAM, WELL, LEED, Green Globes, Living Building Challenge, and HQE [1] assess environmental performance. Materials in green buildings are chosen based on local availability, environmental impact, and regional suitability. Common materials include wood, straw bales, and hempcrete, which are valued for insulation and sustainability. Earth-based materials like adobe and cob are favored for their natural thermal properties in suitable climates. At the same time, bamboo is popular in tropical areas due to its rapid growth and strength. Expanded perlite (EP), widely used in the U.S. and Canada for insulation, is less common globally due to its energy-intensive processing (expansion). Due to these processes, it is also questionable whether it is an all-natural material. However, interest in this material continues to grow due to its unifying features, both in construction and thermally and acoustically insulating material.

EP is still uncommon in Poland. Due to the lack of indigenous deposits, the perlite supply comes from imports from Hungary, Slovakia, and Germany [2]. The perlite is a metamorphic eruptive rock consisting of acid volcanic rhyolite glass, containing 2–5% of water closed in the hardened and weathered lava [3]. Its deposits are practically unlimited [4]. From a chemical point of view, the perlite is a hydrous sodium potassium aluminum silicate, containing mainly silica SiO_2_ (65–75%) and aluminum oxide Al_2_O_3_ (10–18%) as well as sodium and potassium oxides K_2_O + Na_2_O (6–9%), magnesium and calcium oxides MgO + CaO (2–6%), and iron oxide Fe_2_O_3_ (1–5%) [2,5,6,7,8,9]. The chemical composition of the material varies depending on the source of origin. If rapidly heating to a temperature over 850–870 °C, its grains swell, and the bound water abruptly evaporates, making numerous bubbles. During heating, the perlite irreversibly changes its structure—empty, glazed, irregularly shaped bubbles occur inside, which contain the closed air. This process is called expansion (swelling), and the obtained product is expanded perlite (EP). The EP is highly fireproof, sound-absorbing, and resistant to humidity and temperature changes. Moreover, it is resistant to microorganisms like fungi and algae. EP is used in various areas, such as agriculture, medicine, the chemical industry, and construction materials [4]. It is applied as a cultivation base for flowers and vegetables in horticulture because its outstanding porosity contributes to water absorption and dissolved nutrition substances, which are then slowly freed in dry and warm weather [10]. Owing to the containment of significant amounts of air inside the grains and its good capillary properties, the EP mixed with soil is lighter, it aerates, and it opens it. For this reason, the EP has robust applications in many countries for environmental protection due to surface modifications. The EP with the surface coated by hydrophobizing materials absorbs naphtha, gasoline, oils, fats, greases, nonpolar solvents, and all other ‘grease’ oil-derivative liquids on soil and water.

The primary consumer of EP in the Polish market is the building material industry, which uses almost 90% of its total supply in the form of thermal insulating material—either as a loose powder or, more commonly, as a component of dry mortars, concrete mix (so-called perlite concrete), plaster, topping, or adhesive. Thermal conductivity for the EP can vary depending on bulk density, moisture content, particle size, pore structure, and temperature. Still, on average, this value is similar to the thermal conductivity of insulating materials, e.g., Styrofoam and mineral wool. The thermal conductivity for the EP is equal from 0.034 to 0.04 W·m^−1^ K^−1^ [11] for a bulk density of 90 kg·m^−3^ at room temperature 24 °C to 0.059 W·m^−1^ K^−1^ [12,13]. The specific heat of EP is around 837 J·kg^−1^ °C^−1^ [9].

Moreover, it can be used for the manufacturing of ceramic insulating forms resistant to extreme thermal conditions, both in low and high temperatures (chimneys, refrigeration plants, fireproof protections, smoke ducts, and others) because the temperature range of its application amounts from −200 °C to +900 °C. Due to its high sound absorbability, it is used as a component of ceilings, acoustic shields, and noise barriers in concert halls [12].

Usually, during perlite concrete block (PPC) production, sand in the concrete is replaced by the EP. The exchange of the classical sand for the EP evokes essential changes in the products’ physical parameters and rheological features—the more significant the change, the more sand was replaced with EP. In general, the increase in the volumetric share of the perlite at the expense of the sand evokes, unfortunately, a decrease in strength parameters; however, in return, it improves the thermal insulating capacity, fireproofing, and lightness of products [14,15].

Many researchers have studied the characteristic properties of the EP and its use as a construction material. The use of the EP as a thermal insulator in lightweight concrete and brick production was examined, for example, in [16,17,18,19]. Apart from the thermal insulating properties, the influence of EP use on other physical and mechanical properties, such as compressive strength, has also been investigated. It can be found in [3,20,21,22,23].

Another issue that should be paid attention to in erecting walls is the possibility of erecting single-layer walls. Single-layer walls are advantageous because they eliminate the need for separate insulation, thereby simplifying and speeding up construction. This is particularly beneficial in colder climates like Poland. Typically, achieving this requires thicker insulation, which increases costs. Single-layer walls, often over 36 cm thick, offer high durability and ease of construction, only requiring plastering on both sides. However, strict insulation regulations limit the materials suitable for single-layer walls to those with structural and insulating properties, such as autoclaved aerated concrete and porous ceramics. The minimum compressive strength is equal to 1.5 MPa for dry density classes 300, 350, and 400 kg∙m^−3^; 2.5 MPa for dry density classes 450, 500, and 550 kg∙m^−3^; 3 MPa for dry density classes 600 and 650 kg∙m^−3^; 4 MPa for dry density classes 700 and 750 kg∙m^−3^; and 5 MPa for dry density classes 800, 900, and 1000 kg∙m^−3^ [24]. Classifications according to dry density and compressive strength are in Table 1.

The above proved that there is vast innovative potential to be unlocked in pure perlite concrete blocks (PPC), and it is worth carrying out scientific investigations to create a material that has never been seen before on the market and unifies all crucial features, i.e., mechanical strength, heat, acoustic insulating capacity, environmental performance, and economic efficiency.

The research of this article focuses on experimentally determining the mechanical parameters of samples of perlite concrete blocks with 100% EP as aggregate to confirm the thesis that this product will meet the requirements for load-bearing walls in single-family buildings. Samples were compacted with different speeds of displacement and up to various forces, and it was investigated how the compressive strength of the samples changed after 180 days of exposure to outdoor conditions. Additionally, it was investigated how contact with the ground affects the compressive strength of the samples after 270 days of being in outdoor conditions.

## 2. Materials and Methods

### 2.1. Expanded Perlite

**Figure 1:**
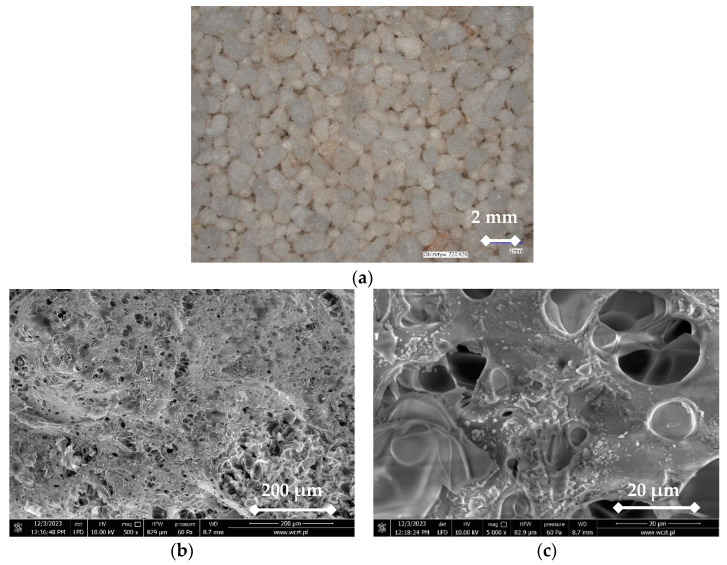

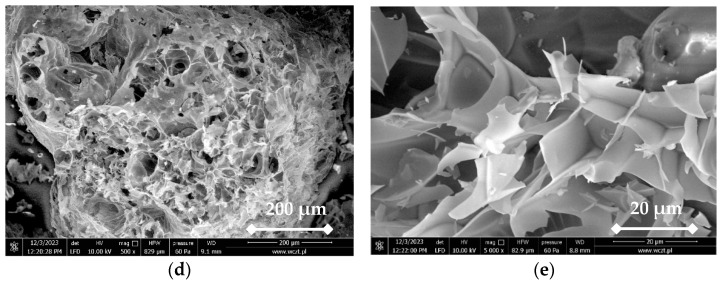
Images of EP: (**a**) Digital image from KEYENCE VHX 6000 (KEYENCE, Mechelen, Belgium) at a magnitude of ×20; SEM images (**b**,**d**) at a magnitude of ×500; SEM images (**c**,**e**) at a magnitude of ×5000 [own study]. The exact concentrations of elements in EP can vary depending on the geographical source, as perlite deposits from different regions may have slightly different chemical compositions. Generally, all EP contained mainly oxygen, silicon, and aluminum, and a smaller amount of iron, potassium, sodium, and calcium. EP mined in Indonesia and analyzed in [25] additionally contained sulfur, magnesium, manganese, titanium, and phosphorus. Sulfur was also contained in EP from Australia [9] and China [5]. On the other hand, manganese was contained in EP from Yemen [26] and Greece [6]. EP mined in the US [8] did not contain titanium.

Expanded perlite class II from one of the Polish distributors was used (Figure 1a). The SEM images are shown in Figure 1b–e. The morphology observation in SEM analysis confirms that the EP is amorphous. Particles can vary in size, typically ranging from a few micrometers to several millimeters, and are generally irregular, having rough surface texture (see Figure 1a). Some SEM images show that EPs have foamy/spongy shapes (see Figure 1b,c), but flake shapes were also observed (see Figure 1d,e). This is confirmed by studies by other researchers, e.g., [7]. The reason for flake-shaped EP geometries in the SEM images might be the processing conditions, such as sieving in industrial manufacturing and pulverization carried out in the laboratory.

**Figure 2:**
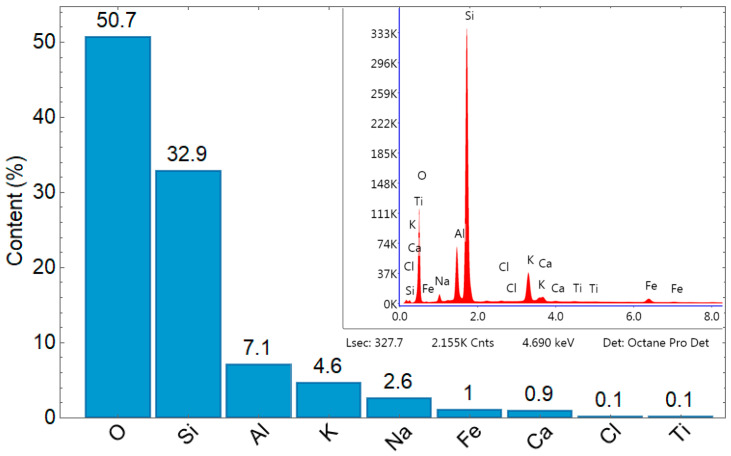
Elemental content by weight in % of EP [own study].

The EP under consideration is characterized by high oxygen and silicon contents, representing 50.7% and 32.9% of the mass, respectively. Aluminum and potassium are the third and fourth most common components, comprising 7.1% and 4.6%, respectively, while sodium, iron, calcium, chlorine, and titanium are in smaller quantities. Similar composition of EPs has been analyzed in [7]. Figure 2 includes the EDX analysis graph where the horizontal axis shows the energy in keV and the vertical axis signifies the intensity count with the removed unimportant part of this graph.

Table 2 presents the results of the measurements of the bulk and skeletal densities of EP, which was the subject of the research. The grain size of EP is shown in Table 3.

A particle’s true or specific surface area, including surface irregularities and pore walls, is determined at an atomic level by the adsorption of an unreactive gas and measured using the Brunauer–Emmett–Teller (BET) theory. BET Surface Area Analysis was estimated at 77 K degrees under liquid nitrogen. Before measurement, samples of EP were degassed under vacuum at 120 °C for 2 h. Typically, the BET surface area of EP is between 1 and 3 m^2^ g^−1^ [7]. On the other hand, surface areas greater than 5 m^2^ g^−1^ were also reported in the literature due to different processing, physical conditions, and origin of mines [7]. The expanded perlite under analysis had a BET total surface area equal to 3.0202 ± 0.0167 m^2^ g^−1^.

The skeletal density was examined with an Accupyc II 1345 helium pycnometer (Micromeritics, Norcross, GA, USA). In measurements, a 10 cm^3^ chamber was used, ten rinses of the sample were applied, and the result was determined from ten measurements. The bulk density, both in loose and compacted form, was determined based on the EN 1097-3 standard [27]. The results obtained are in line with the values known from the literature. The bulk density (loose) of expanded perlite typically ranges from 25 to 400 kg·m^−3^ [17,28,29] and depends on the degree of expansion, particle size, quality, and moisture content [30]. The most crucial process is the expansion process—greater expansion results in lower density. For example, EPs analyzed in [7], despite the same mining area, similar chemical composition, and SEM images, were characterized by different actual (skeletal) density values from 0.84 g·cm^−3^ to 2.45 g·cm^−3^. It is important to note that the density of EP is significantly lower than that of unexpanded (raw) perlite, which typically has a density of around 956 to 1342 kg·m^−3^ [31]. The low density of EP contributes to its lightweight nature and excellent insulation properties, making it suitable for various applications such as construction, horticulture, filtration, and industrial uses.

### 2.2. Portland Cement

**Figure 3:**
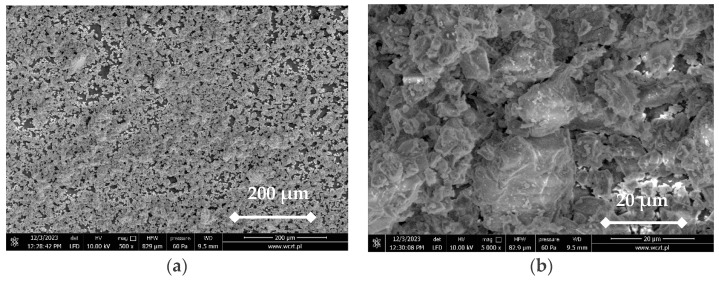
SEM images of PC: (**a**) magnitude ×500; (**b**) magnitude ×5000 [own study].

**Figure 4:**
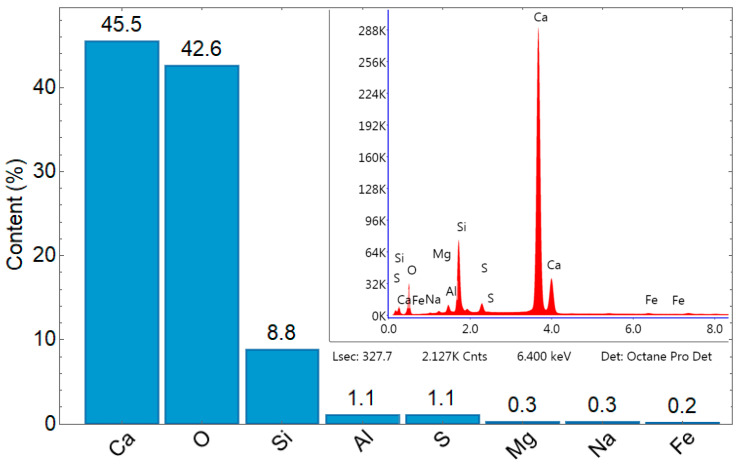
Elemental content by weight in % of PC [own study].

White Portland cement CEM I 52.5 R marked in this article as PC was used, meeting the requirements of the EN-197-1 standard [32]. Figure 3 presents an SEM image in 500 magnitudes, and Figure 4 lists the chemical composition of PC. The dominant elements are oxygen and calcium, with concentrations of 45.5% and 42.6%, respectively. Silicon is present at 8.8%, while aluminum, sulfur, magnesium, sodium, and iron occur in smaller proportions. Figure 4 includes the EDX analysis graph, where the horizontal axis shows the energy in keV, and the vertical axis signifies the intensity count, with the unimportant part of this graph removed.

Before using a PC, tests were carried out on its bending and compressive strengths. Strength tests of cement paste, i.e., bending strength and compressive strength, were carried out based on PN-EN 196-1 [33].

To carry out the tests, sets of three specimens in the shape of cuboidal beams with dimensions of 40 mm × 40 mm × 160 mm were prepared. Each portion of the three beams consisted of (450 ± 2) g of cement, (1350 ± 5) g of sand, and (225 ± 1) g of water. EN 196-1 indicates that CEN standard sand from different sources and countries can be used, provided the cement strength results are not significantly different from those obtained using CEN standard sand. This study used standard sand from KWARCMIX to prepare cement paste.

The beams were unmolded after 48 h. Tests on the strength of beams of different ages were performed with the following tolerance: 48 h ± 30 min, 72 h ± 45 min, 7 days ± 2 h, 28 days ± 8 h. A three-point loading method was used for bending strength testing. A special machine insert was used for this purpose. Beams were placed in the machine, with the side surface on the support rollers, so its longitudinal axis was perpendicular to the support rollers. Using a load roller, a load was applied vertically to the opposite side surface of the beam by uniformly increasing the pressure by (50 ± 10) N∙s^−1^ until it broke. The beam halves were covered with a damp cloth until a compression test was performed.

Bending strength, *R_f_*, was calculated according to the formula

(1) $$R_{f}=\frac{1.5\;\cdot \;F_{f}\;\cdot \;l}{b^{3}}$$

where:

*b*—lateral length of the beam section (mm);

*F_f_*—breaking load at the center of the beam (N);

*l*—distance between supports (mm).

The result of the bending strength test is the arithmetic mean of three individual results expressed with an accuracy of 0.1 MPa.

Compressive strength testing was performed on the halves of beams broken during bending strength testing. A special machine insert was used for this purpose. Each half of the beam was tested by loading its side surfaces. The beam halves were placed laterally in the center of the plate, with an accuracy of ±0.5 mm, and in the longitudinal direction so that the faces of the beam protruded about 10 mm beyond the plates or auxiliary plates.

The compressive strength, *R_c_*, was calculated according to the formula

(2) $$R_{c}=\frac{F_{c}}{1600}$$

where:

*F_c_*—the highest load when crushing the specimen (N);

1600—an area of plates or auxiliary plates (40 × 40) (mm^2^).

The compressive strength test result is the arithmetic average of six individual results, expressed with an accuracy of at least 0.1 MPa. The average value of bending and compressive strength of PC after 28 days is shown in Table 4, comparing these results with the manufacturer’s declaration.

### 2.3. Mixture of Perlite Concrete (PPC)

**Figure 5:**
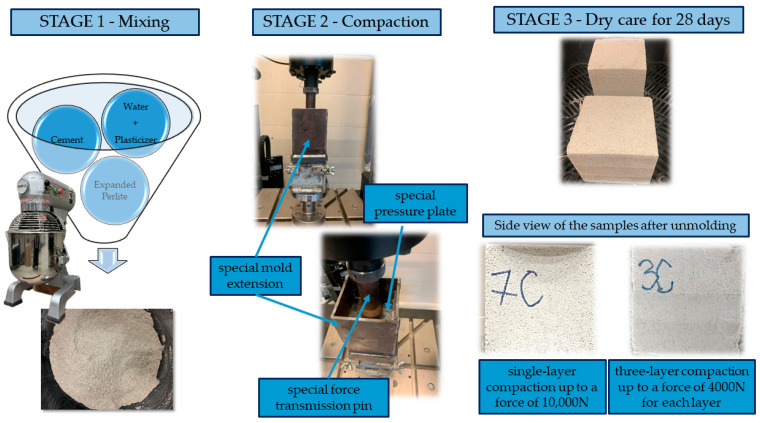
Stages of preparation of the samples.

To produce pure perlite concrete (PPC) samples, EP was mixed with PC and water (Figure 5). First, water with a plasticizer was mixed with cement, then, EP was gradually added. In the next step, the samples were compacted. The MasterX-SEED BASF plasticizer was used. Its task was to improve the value of both the early and final compressive strength of PPC, which, according to specifications, optimizes the use of raw materials and benefits sustainable construction.

The composition of the mixture is shown in Table 5, while the procedure for mixing the ingredients is outlined below. The composition of the mixture was determined based on the literature analysis and pilot studies. It was essential to determine the water content.

Each batch of the PPC mixture was mixed mechanically with a mixer (Figure 5).

The mixing procedure was as follows:

- Portions of water, plasticizer, PC, and EP were prepared, respectively.
- Water with plasticizer and PC were transferred to the bowl, avoiding losses.
- After the PC and water came into contact, the mixer was started for 30 s at a low speed of 110 RPM, and the so-called “zero time” was recorded.
- EP was dosed for another 60 s while mixing at a low speed of 110 RPM.
- After all the EP was in a bowl, the mixer was switched to a higher speed of 178 RPM, and mixing continued for another 60 s.
- Mixing was stopped for 60 s, and during the first 30 s, a plastic scraper was used to pick up the mixture that had adhered to the bowl.
- Mixing was continued for another 60 s, keeping the highest speed of 355 RPM.
- Mixing was stopped for 60 s, and during the first 30 s, a plastic scraper was used to pick up the mortar that had adhered to the bowl.
- Mixing was continued for another 60 s, keeping the highest speed of 355 RPM.
- The coordination of the mixing steps refers to the periods when the mixer power is on/off and was maintained within ±5 s.

Cubic samples (100 × 100 × 100 mm) were formed by compaction immediately after mixture preparation. The samples were compacted at a constant displacement speed of 15 mm per minute or 150 mm per minute until the force reached 4000 N or 10,000 N. In addition, two compaction methods were used—single-layer or three-layer compaction. The amount of mixture needed was taken by weight. The appropriate amount of mixture for a given type of compaction was determined by trial and error. To ensure proper compaction, a special extension was designed for the steel mold, and a special pressure plate was used to transmit force to the surface so that compaction would be even.

Figure 5 shows the mixer, the appearance of the mixture in the preparation stage, the appearance of the designed extension for preparing samples during compaction, and the single-layer and three-layer samples after removing them from the molds.

After compaction, samples were stored in a climatic chamber for dry conditions for 14 and 28 days. The temperature was equal to 25 °C, and the humidity was equal to 100%.

### 2.4. Compressive Strength

The compressive strength tests of the PPC samples were performed according to the standard EN 12390-3 [33,34]. Investigations of the maximum force that an individual sample can withstand were conducted in the INSTRON 8806 hydraulic testing machine (Norwood, MA, USA). After carefully cleaning the sample surfaces, the sample was placed centrally on the cleaned surface of the machine pressure plates and loaded with a constant displacement speed of 15 mm per minute, like with concrete samples [35]. The compressive strength value was determined with a precision of 0.01 MPa.

In the specification of masonry elements, the compressive strength of PPC should not be less than 1.5 MPa at a moisture content of 6 ± 2% for dry density class 400 [24]. Analysis of the relationship between dry density and compressive strength confirmed that PPC blocks allow the erection of load-bearing and non-bearing wall connections.

After dry curing for 14 or 28 days, the weight of the sample with an accuracy of 1 g and the dimensions with an accuracy of 1 mm were measured. Then, the samples were rotated by 90 degrees from the forming position, and the compressive strength test was conducted. The load at which the cubes failed was noted, and the compressive strength was calculated. After the tests, the type of failure and appearance of cracks were also reported.

## 3. Results and Discussion

### 3.1. Effect of Displacement Speed During Sample Compaction on Compressive Strength

The first stage of the study was to determine the proper displacement speed under loading needed to compact the PPC samples. For this purpose, two sets of PPC samples were prepared—one compacted slowly with a displacement speed of 15 mm per minute and another compacted quickly at a displacement of 150 mm per minute. At this stage, both sets of samples with a given displacement speed were compacted until the loading force values reached 4000 N. The amount of mixture needed was determined by weight by trial and error.

**Figure 6:**
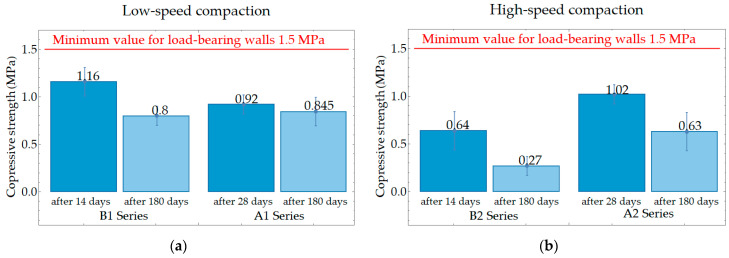
Average compressive strength for samples compacted up to a force of 4000 N: (**a**) low-speed compaction; (**b**) high-speed compaction.

Compressive strength was tested after 14 (series B) and 28 (series A) days on PPC samples that were stored in dry laboratory conditions and after 180 days on samples that were seasoned outdoors (from 30 September 2021 to 30 May 2022). Figure 6 shows a comparison of compressive strength results. In the graphs in Figure 6 and the next compressive strength diagrams, the red line represents the minimum value of compressive strength required for the material to be used to erect the load-bearing walls of the building, i.e., 1.5 MPa when a class of density is equal to 300, 350, or 400. A load-bearing wall is an active structural element of a building that holds the weight of the elements above it by conducting its weight to a foundation structure below it.

**Figure 7:**
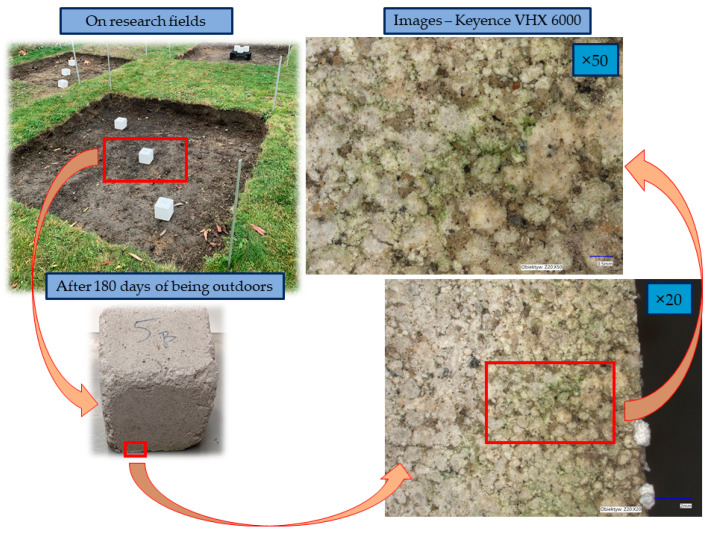
A collection of images presenting the surface of the sample after 180 days of being outdoors.

All sets of samples showed a decrease in compressive strength after a period of 180 days of being outdoors. The decrease for samples compacted with a higher displacement speed was 57.8% for the samples that were cured under laboratory conditions for 14 days before being exposed to the outdoor conditions. The decrease was 38.2% for the samples that were cured under laboratory conditions for 28 days before being exposed to the outdoor conditions. The decrease in the strength of the samples compacted at low speed was less, amounting to 31% for the samples that were cured under laboratory conditions for 14 days before being exposed to the outdoors, and 8.2% for the samples that were cured under laboratory conditions for 28 days before being exposed to the outdoors. Thus, it can be concluded that compaction at a lower displacement rate guarantees higher values of compressive strength and greater stability of compressive strength; however, compressive strength values greater than 1.5 MPa were not achieved for any of these series. Figure 7 presents a view under a digital microscope of KEYENCE VHX 6000 of the sample after 180 days of being outdoors in the research field. The research field was 2 × 2 m and had a 25 cm top layer of soil removed. Algae that have located themselves on the soggy walls of the sample can be seen. The saturated area is an ideal medium for algae and is also the cause of the possibility of the appearance of fungus or moss. This is a rationale for treating the surface of PPC with a suitable protective agent.

### 3.2. Effect of Force up to Which Samples Were Compacted on Compressive Strength

**Figure 8:**
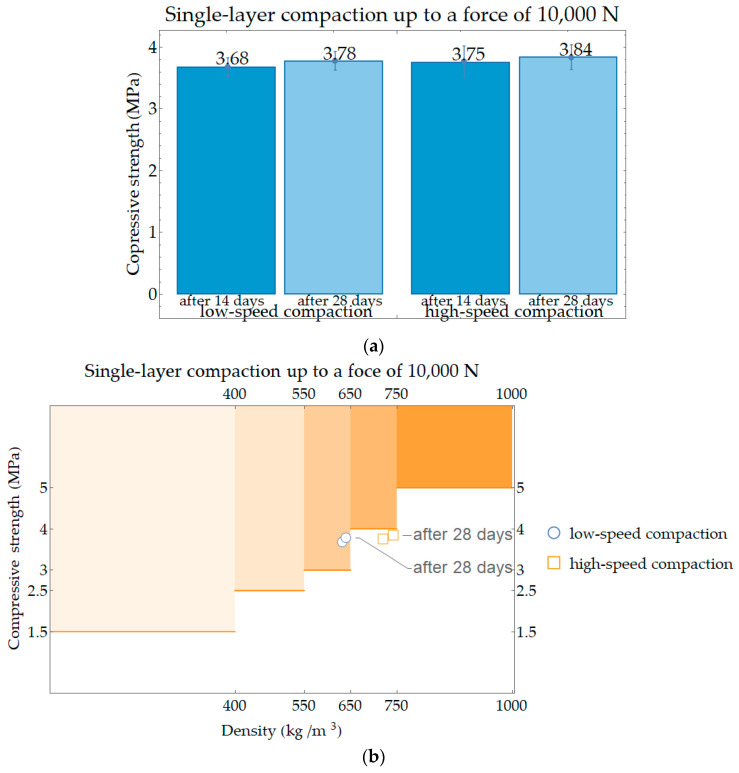
Single-layer compaction up to a force of 10,000 N under low-speed and high-speed compaction: (**a**) average compressive strength; (**b**) diagram for investigating the minimum compressive strength class of elements due to gross dry density class.

In the next stage of this research, the correlation was investigated between the speed of displacement during forcing and the value of force up to which the PPC samples were compacted. The compressive strength was measured after 14 and 28 days for the samples compacted with the speed of displacement equal to 15 mm per minute, and 150 mm per minute up to the force of 10,000 N. The results are presented in Figure 8a. At the same time, the results obtained were investigated to see how they relate to the requirements for minimum compressive strength to density. For this purpose, in Figure 8b, a diagram was created on the basis of the data in Table 1, where individual areas in the orange shade were marked with areas belonging to a given strength class in relation to the density class. The diagram should be read in such a way that if the point is located in the area of the orange boxes, the requirements for lightweight aggregate concrete as load-bearing walls can be met.

**Figure 9:**
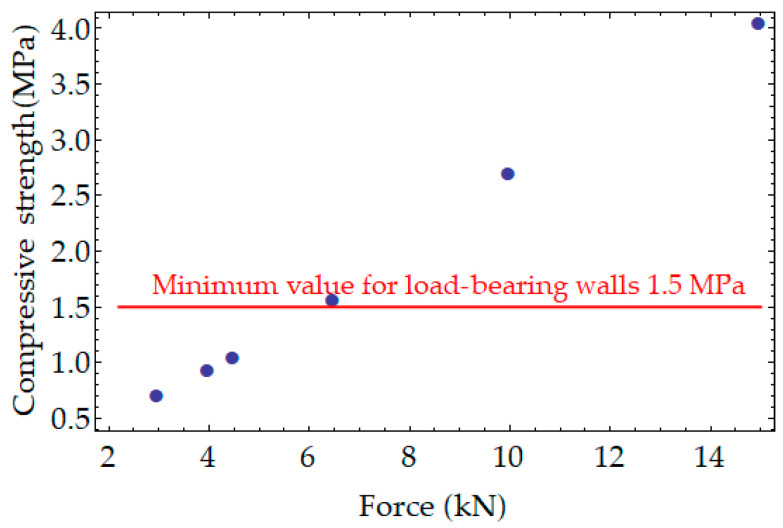
Graph to find the proper force up to which samples of PPC should be compacted.

The value of force up to which the PPC samples were compacted in this stage of research was determined from an additional test, where compressive strength after 28 days was investigated for samples that were compacted with a speed of displacement equal to 15 mm per minute up to forces of 3000 N, 4000 N, 4500 N, 6500 N, 10,000 N, and 15,000 N (Figure 9). It was decided that the force of 10,000 N would be proper, taking into account the compressive strength value after 28 days and the fact that higher loading speed and external conditions can reduce the value of the compressive strength of PPC samples.

**Figure 10:**
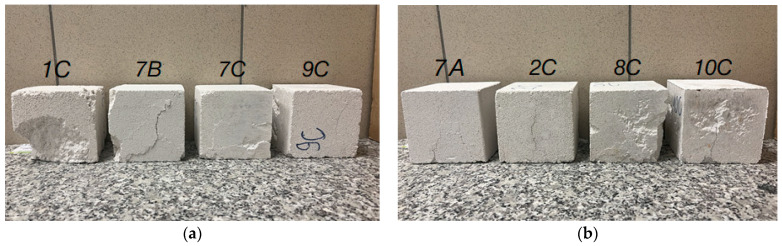
Types of destruction of solid (single-layer) PPC samples after compressive strength tests were conducted (**a**) after 14 days of being stored in dry laboratory conditions; (**b**) after 28 days of being stored in dry laboratory conditions, where 1C and 7A represent low-speed compaction up to a force of 4000 N, 7B and 2C represent high-speed compaction up to a force of 4000 N, 7C and 8C represent low-speed compaction up to a force of 10,000 N, and 9C and 10C represent high-speed compaction up to a force of 10,000 N.

The results of the compressive strengths obtained at this stage of the research were related to the results presented in Figure 6. At the higher force to which the samples were compacted, the condition regarding the material’s applicability as a load-bearing wall in a single-family building was met for the low-speed compacted sample; compressive strength values were higher than 3 MPa, which is the minimum value for dry density class 600 to which they belong. A higher speed of displacement during loading affects the results; compressive strength values were lower than 4 MPa for the dry density class to which they belong, i.e., class 700. Types of destruction of single-layer (solid) samples after compressive strength tests conducted after 14 and 28 days of storage in dry laboratory conditions are shown in Figure 10. The samples, in most cases, show a failure character similar to that of concrete samples, i.e., tending toward an hourglass shape, although the cracks often run closer to the center of the specimens.

### 3.3. Effect of the Samples’ Forming Method on Compressive Strength

**Figure 11:**
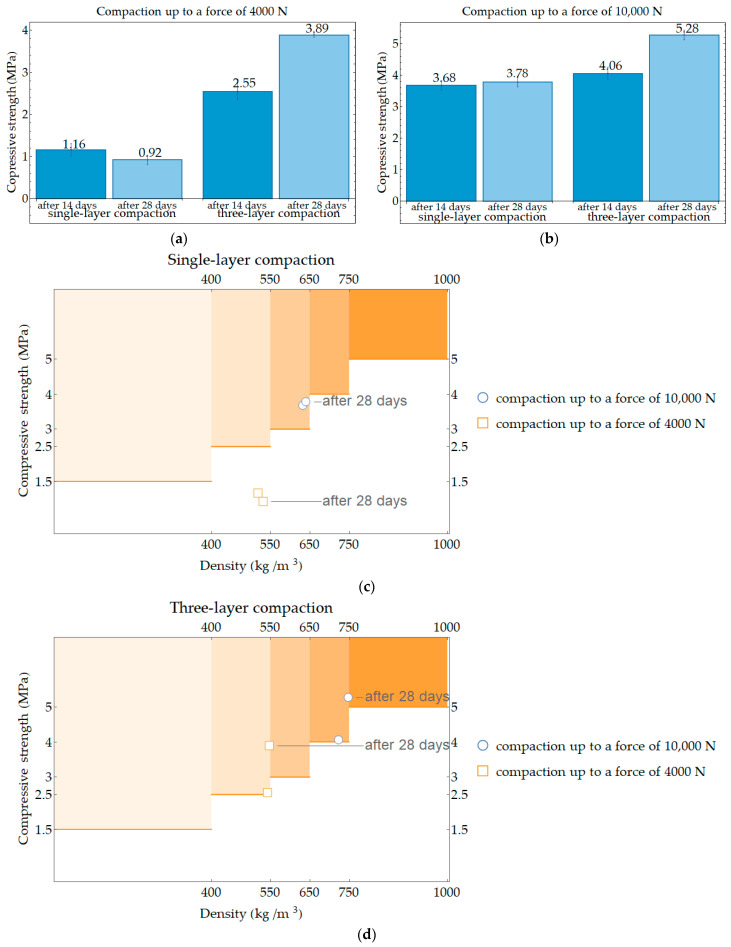
Average compressive strength for PPC samples compacted with a constant speed of displacement at 15 mm per minute (**a**) up to a force of 4000 N, (**b**) up to a force of 10,000 N, and a diagram for investigating the minimum compressive strength class of elements due to gross dry density class; (**c**) for single-layer compaction; (**d**) for three-layer compaction.

Figure 11 shows the compressive strength results of the PPC samples after 14 and 28 days for each compaction method (single-layer or three-layer compaction), depending on the maximum compaction force. Samples were compacted at a displacement speed of 15 mm per minute and loaded to force values up to 4000 N and 10,000 N. Only compaction in three layers provided minimum compressive strength during loading up to the force of 4000 N; yet, results are still on the border of the range of correctness. When compaction up to the force of 10,000 N provided minimum compressive strength regardless of the type of sample molding, the dry density value was also close to the limit of the range of correctness.

The main conclusions from this analysis are as follows: the tested compressive strength does not meet the minimum condition for the material to be the load-bearing wall of the building, i.e., when solid samples (i.e., from single-layer compaction) are compacted up to a force of 4000 N; this force also only provides adequate compressive strength after 28 days of curing when compaction was conducted in three layers (min. compressive strength is equal 2.5 MPa because the class of dry density is 550 kg∙m^−3^). Compaction up to a force of 10,000 N ensures adequate compressive strength is achieved already with single-layer compaction—min. compressive strength is equal to 2.5 MPa because the class of dry density is 550 kg∙m^−3^. Compaction up to a force of 10,000 N is recommended for further research.

**Figure 12:**
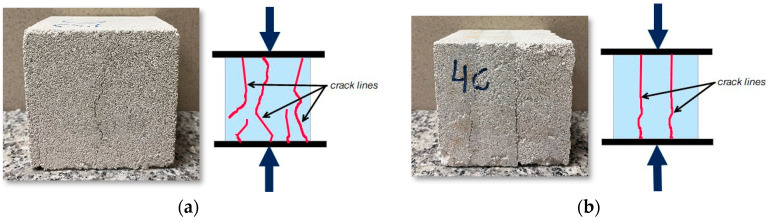
Types of destruction (**a**) of a single-layer compacted sample, and (**b**) of a three-layer compacted sample.

After the compressive strength test was completed, the type of specimen destruction was investigated. Figure 12 shows the destruction of the samples. The crack lines in solid samples are similar to those observed in Figure 10 and those observed in ordinary concrete samples. The crack lines in samples compacted with three layers followed the layers and split the sample into three pieces.

**Figure 13:**
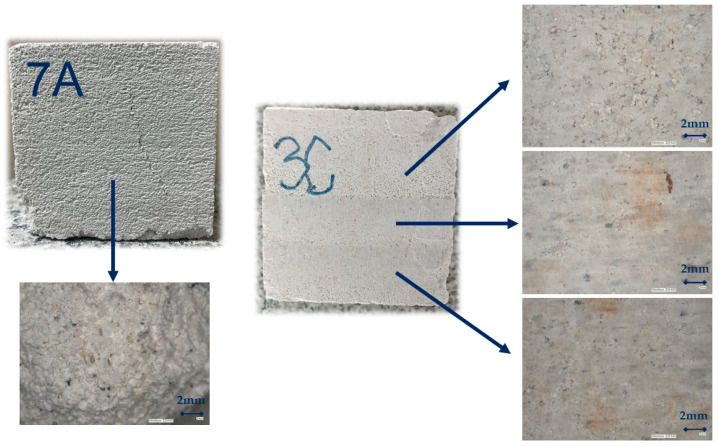
The collection of images presents a comparison of the surface of a solid with a three-layer compacted sample. The magnitude was equal to 20. The zoomed images came from KEYENCE VHX 6000.

Figure 13 compares digital imaging of solid and three-layer compacted samples.

Considering the results from compressive strength and observations of types of failure, it was determined that in the next research stage, the PPC samples should be compacted at a displacement speed of 15 mm per minute in a single layer with a force up to 10,000 N.

### 3.4. Effect of External Conditions on Compressive Strength

**Figure 14:**
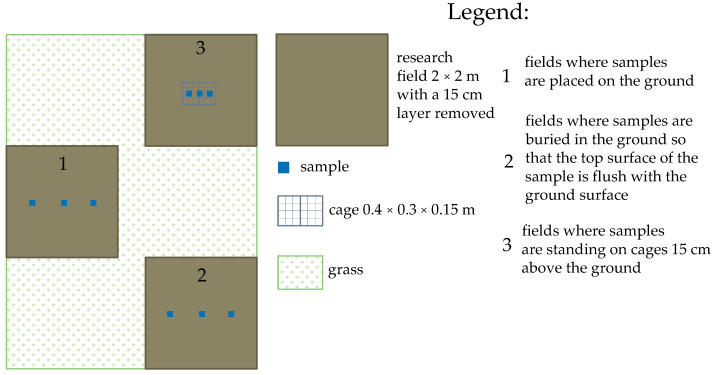
Scheme of research fields.

**Figure 15:**
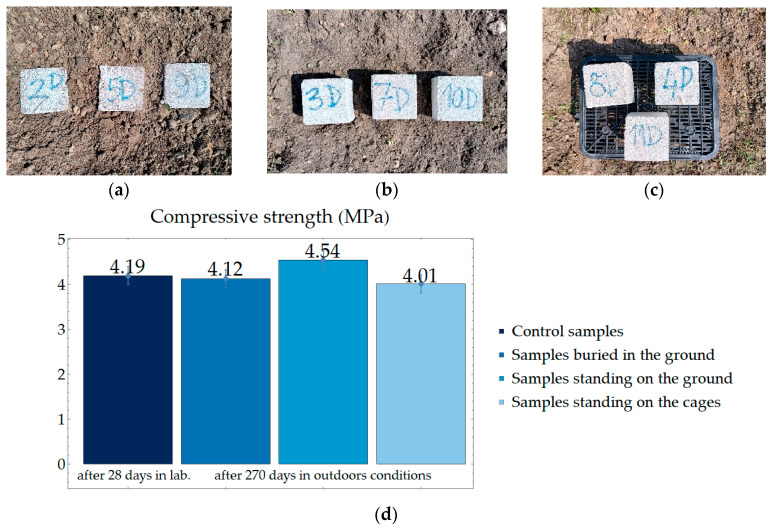

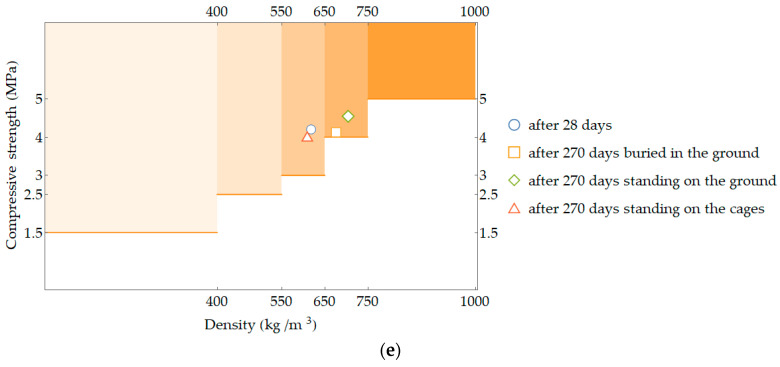
Average compressive strength of samples compacted up to a force of 1000 N with a constant displacement of 15 mm per min. (**a**) Samples buried in the ground; (**b**) samples that stood on the ground; (**c**) samples that stood on the box; (**d**) graph for compressive strength; (**e**) diagram for investigating the minimum compressive strength class of elements due to gross dry density class.

In the next stage of the research, it was investigated how the compressive strength of the samples changed after 270 days (from 28 July 2022 to 24 April 2023) of exposure to outdoor conditions. The period included winter. Additionally, it was investigated how contact with the ground affects the compressive strength of the PPC samples. Some of the samples were buried in the ground, some on the ground, and some on boxes for them to not come into contact with the ground. Figure 14 presents a scheme of research fields. Compressive strength results for samples from outdoor conditions compared with the value of the control sample are presented in Figure 15.

After 270 days of being outdoors, the samples buried and stood on boxes slightly decreased their compressive strength compared to the control samples. However, samples that stood on the ground became stronger after 270 days outdoors. The reason for this was because of the optimal water conditions for these samples. Samples buried in the ground had too much contact with wet ground, and those standing on the cage were exposed to weathering.

**Figure 16:**
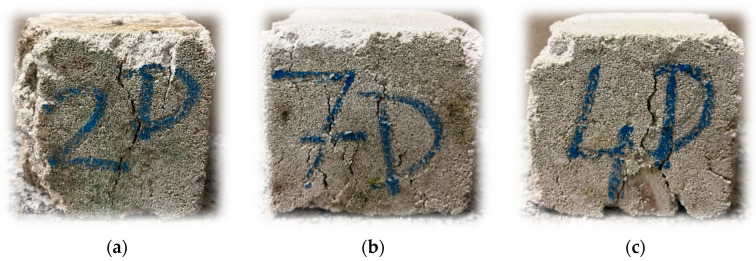
Samples that have been outdoors after strength tests: (**a**) samples buried in the ground; (**b**) samples that stood on the ground; (**c**) samples that stood on the boxes.

The samples’ demolished look is shown in Figure 16. The samples have crushed edges and are covered with algae. The cracks run longitudinally, arranging themselves mainly in the center of the samples.

**Figure 17:**
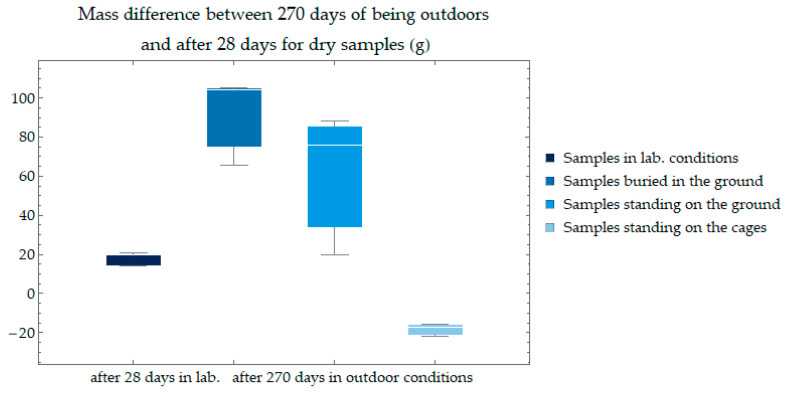
Change in the samples’ masses. Single-layer compaction up to a force of 10,000 N.

Samples that were buried and stood on the ground after being outdoors increased in weight; samples that stood on boxes lost weight. The difference in the weights of the samples was determined as the difference in the weight of the sample after 28 days of seasoning under laboratory conditions and the weight after another 270 days under outdoor conditions. The masses were determined for samples dried in an oven at 105 degrees Celsius for a period of 24 h. The change in masses is shown in Figure 17. A large coefficient of variation characterized the results. The most significant variation in weight change results was recorded for samples standing on the ground. The smallest variation was for control and cage-standing samples.

### 3.5. Comparison of Results with the Literature

Increasing the EP content in blocks, bricks, or concrete reduces their density and compressive strength. When EP replaces 80% of the aggregate, concrete density drops significantly, categorizing it as lightweight. For instance, concrete density can decrease from 1710 to 560 kg·m^−3^, with a corresponding drop in compressive strength from 28.8 MPa to 3.4 MPa after 28 days [3]. Similar results were found by other researchers [20], showing a decrease in density from 1937 to 673 kg·m^−3^ and compressive strength from 28.8 MPa to 1.1 MPa. With 100% EP, density falls to 392 kg·m^−3^ and compressive strength to 0.1 MPa. Carabba et al. [36] confirmed these trends, showing that replacing all sand with EP results in an average compressive strength of 3.3 MPa. As mentioned above, most studies involve perlite concrete, where a certain percentage of aggregate or cement is replaced with expanded perlite [37,38,39]. There are few works on analyzing pure perlite concrete (PPC), a product where 100% of the aggregate has been replaced with EP. Results presented in [40] show that the density of perlite concrete with 100% EP is 649.88–705.14 kg·m^−3^, and compressive strength after 28 days is 4.99–6.10 MPa. Relating this to the standard [24], the requirement for minimum compressive strength, i.e., 4 MPa, is met. These results are similar to those obtained in the current study.

## 4. Conclusions

Based on the research, the following conclusions can be said:

- The designed extension for the mold with a special pressure plate for compaction samples fulfills the task.
- The proposed mixing procedure ensures the accurate mixing of ingredients, but there is a need to research the relationship between water, cement, and EP to find the optimal mixture. This issue can be analyzed based on [41] or by using machine learning [42].
- The optimal compaction method was determined as follows:
  o Single-layer compaction is better. However, further research on structures with three or more layers should be conducted.
  o PPC samples should be compacted at a displacement speed of 15 mm per minute.
  o Compaction up to a force of 10,000 N ensures adequate compressive strength to erect load-bearing single-layer walls from PPC.
- The higher the density, the higher (favorable) the compressive strength.
- Samples seasoned in outdoor conditions lose compressive strength except for those that stand on the ground. This means that the strength of PPC is greatly affected by outdoor conditions; this should be kept in mind when erecting walls from this material.

## 5. Future Research

The main goal of future research on using expanded perlite concrete (PPC) in construction is to modify PPC properties to enable the construction of external single-layer walls that meet stringent energy and structural requirements. Specifically, this research aims to develop PPC blocks that achieve proper compressive strength, making them suitable for load-bearing applications and providing a heat transfer coefficient (U-value) of less than 0.2 W∙m^−2^ K^−1^. This thermal performance target considers not only the PPC block itself but also the addition of external plaster finishes and the thermal resistance at both the internal and external wall surfaces.

Achieving these targets would make PPC blocks a viable option for energy-efficient wall systems, eliminating the need for additional insulation layers, thus simplifying construction processes and reducing material costs. Future research could explore optimizing the pore structure of perlite within PPC to increase its insulative capacity without compromising its strength. Research is still necessary to optimize the perlite concrete mixture, i.e., the appropriate share of EP with a specific grain size, cement, and water. The key is to increase moisture resistance.

In addition, research could investigate sustainable methods for the expansion process of perlite to make it more energy-efficient and environmentally friendly. This could increase the appeal of PPC as a natural and low-impact building material that aligns with modern green building standards, potentially expanding its use in sustainable construction practices worldwide.

## Figures and Tables

**Table 1:** Classification according to dry density and average compressive strength [24].

Class of Density	Range of Density in Air-Dry Conditions kg∙m^−3^	Class of Compressive Strength	Average Compressive Strength N∙mm^−2^
300	From 250 to 300	1.5	1.5
350	>300 and ≤350	2	2
400	>350 and ≤400	2.5	2.5
450	>400 and ≤450	3	3
500	>450 and ≤500	3.5	3.5
550	>500 and ≤550	4	4
600	>550 and ≤600	4.5	4.5
650	>600 and ≤650	5	5
700	>650 and ≤700	6	6
750	>700 and ≤750	7	7
800	>750 and ≤800		
900	>800 and ≤900		
100	>900 and ≤1000		
The dry density is determined with an accuracy of 5 kg∙m^−3^			
**Class of density**		**Minimum compressive strength N/mm^2^**	
300; 350; 400		1.5	
450; 500; 550		2.5	
600; 650		3	
700; 750		4	
800; 900; 1000		5	

**Table 2:** Skeletal and bulk density of expanded perlite from one of the Polish distributors [own study].

Skeletal Density (g·cm^−3^)	Bulk Density (g·cm^−3^)	
	Loose	Compacted
1.4717	0.0824	0.09601

**Table 3:** Summary of fractional content [own study].

**Aggregate Fractions (mm)**	0–0.063	0.063–0.1	0.1–0.25	0.35–0.5	0.5–1	1–2
**Percentage of fractions in the aggregate pile (%)**	1.5	2.3	7	8.6	31.4	49.2

**Table 4:** The average value of bending and compressive strength of PC after 28 days.

Bending Strength (MPa)		Compressive Strength (MPa)	
In Measurements	From the Manufacturer’s Declaration	In Measurements	From the Manufacturer’s Declaration
9.8	-	72.4	≥52.5

**Table 5:** Composition of the PPC mixer.

Expanded Perlite	Cement	Water	Plasticizer
125 L	25 kg	22.5 L	100 mL

## Data Availability

The original contributions presented in this study are included in the article. Further inquiries can be directed to the corresponding author.

## References

[1] Mazur Ł., Szlachetka O., Jeleniewicz K., Piotrowski M. (2024). External Wall Systems in Passive House Standard: Material, Thermal and Environmental LCA Analysis. Buildings.

[2] Dzięcioł J., Szlachetka O. (2024). Waste or Raw Material? Perlite-Concrete as Part of a Sustainable Materials Management Process in the Construction Sector. Sustainability.

[3] Jedidi M., Benjeddou O., Soussi C. (2015). Effect of Expanded Perlite Aggregate Dosage on Properties of Lightweight Concrete. Jordan J. Civ. Eng..

[4] Celik A.G., Kilic A.M., Cakal G.O. (2013). Expanded Perlite Aggregate Characterization for Use as a Lightweight Construction Raw Material. Physicochem. Probl. Miner. Process..

[5] Wang X., Wu D., Geng Q., Hou D., Wang M., Li L., Wang P., Chen D., Sun Z. (2021). Characterization of Sustainable Ultra-High Performance Concrete (UHPC) Including Expanded Perlite. Constr. Build. Mater..

[6] Kaufhold S., Reese A., Schwiebacher W., Dohrmann R., Grathoff G.H., Warr L.N., Halisch M., Müller C., Schwarz-Schampera U., Ufer K. (2014). Porosity and Distribution of Water in Perlite from the Island of Milos, Greece. J. Korean Phys. Soc..

[7] Aksoy Ö., Alyamaç E., Mocan M., Sütçü M., Özveren-Uçar N., Seydibeyoğlu M.Ö. (2022). Characterization of Perlite Powders from Izmir, Türkiye Region. Physicochem. Probl. Miner. Process..

[8] Maxim L.D., Niebo R., Mcconnell E.E. (2014). Perlite Toxicology and Epidemiology—A Review. Inhal. Toxicol..

[9] Ariyaratne I.E., Ariyanayagam A., Mahendran M. (2022). Bushfire-Resistant Lightweight Masonry Blocks with Expanded Perlite Aggregate. Fire.

[10] Dzięcioł J., Szlachetka O., Rodrigues Tavares J.M. (2024). From Volcanic Popcorn to the Material of the Future. A Critical Review of Expanded Perlite Applications and Environmental Impacts. Sustainability.

[11] Huang G., Pudasainee D., Gupta R., Liu W.V. (2021). Thermal Properties of Calcium Sulfoaluminate Cement-Based Mortars Incorporated with Expanded Perlite Cured at Cold Temperatures. Constr. Build. Mater..

[12] Burkowicz A. (2016). Perlit Ekspandowany—Materiał Termoizolacyjny Mało Znany w Polsce. Zesz. Nauk. Inst. Gospod. Surowcami Miner. I Energią Pol. Akad. Nauk.

[13] Szymczak-Graczyk A., Gajewska G., Laks I., Kostrzewski W. (2022). Influence of Variable Moisture Conditions on the Value of the Thermal Conductivity of Selected Insulation Materials Used in Passive Buildings. Energies.

[14] Sharook S., Sathyan D., Madhavan M.K. (2020). Thermo-Mechanical and Durability Properties of Expanded Perlite Aggregate Foamed Concrete. Proc. Inst. Civ. Eng. Constr. Mater..

[15] Akyuncu V., Sanliturk F. (2021). Investigation of Physical and Mechanical Properties of Mortars Produced by Polymer Coated Perlite Aggregate. J. Build. Eng..

[16] Polat R., Demirboğa R., Karagöl F. (2018). The Influence of Expanded Perlite Aggregate on Compressive Strength, Linear Autogenous Shrinkage, Restrained Shrinkage, Heat of Hydration of Cement-Based Materials. Struct. Concr..

[17] Topçu I.B., Işikdaǧ B. (2007). Manufacture of High Heat Conductivity Resistant Clay Bricks Containing Perlite. Build. Environ..

[18] Demirboǧa R., Örüng I., Gül R. (2001). Effects of Expanded Perlite Aggregate and Mineral Admixtures on the Compressive Strength of Low-Density Concretes. Cem. Concr. Res..

[19] Lanzón M., García-Ruiz P.A. (2008). Lightweight Cement Mortars: Advantages and Inconveniences of Expanded Perlite and Its Influence on Fresh and Hardened State and Durability. Constr. Build. Mater..

[20] Sengul O., Azizi S., Karaosmanoglu F., Tasdemir M.A. (2011). Effect of Expanded Perlite on the Mechanical Properties and Thermal Conductivity of Lightweight Concrete. Energy Build..

[21] Alyousef R., Benjeddou O., Soussi C., Khadimallah M.A., Jedidi M. (2019). Experimental Study of New Insulation Lightweight Concrete Block Floor Based on Perlite Aggregate, Natural Sand, and Sand Obtained from Marble Waste. Adv. Mater. Sci. Eng..

[22] Khonsari V., Eslami E., Anvari A. Effects of Expanded Perlite Aggregate on the Mechanical Behavior of Lightweight Concrete. Proceedings of the 7th International Conference on Fracture and Mechanics of Concrete & Concrete Structure (FraMCoS-7).

[23] Tapan M., Engin C. (2019). Effect of Expanded Perlite Aggregate Size on Physical and Mechanical Properties of Ultra Lightweight Concrete Produced with Expanded Perlite Aggregate. Period. Polytech. Civ. Eng..

[24] (2015). Specification for Masonry Units—Part 4: Autoclaved Aerated Concrete Masonry Units.

[25] Adi Darmawan D., Wahyudi A., Eric Mamby H., Suherman I. (2021). Characterization of Perlite and Expanded Perlite from West Sumatera, Indonesia. IOP Conf. Ser. Earth Environ. Sci..

[26] Al-Selwi K.M.T. (2023). Occurrences of Perlite Deposits in Yemen. Thamar Univ. J. Nat. Appl. Sci..

[27] (2000). Mechanical Tests and Properties of Aggregate Properties—Determination of Bulk and Voids Properties.

[28] Singh M., Garg M. (1991). Perlite-Based Building Materials—A Review of Current Applications. Constr. Build. Mater..

[29] Koukouzas N.K., Dunham A.C., Scott P.W. (2000). Suitability of Greek perlite for industrial applications. Appl. Earth Sci..

[30] Sodeyama K., Sakka Y., Kamino Y., Seki H. (1999). Preparation of Fine Expanded Perlite. J. Mater. Sci..

[31] Saisuttichai D., Manning D.A.C. (2007). Geochemical Characteristics and Expansion Properties of a Highly Potassic Perlitic Rhyolite from Lopburi, Thailand. Resour. Geol..

[32] (2011). Cement—Part 1: Composition, Specifications and Conformity Criteria for Common Cements.

[33] (2016). Methods of Testing Cement—Part 1: Determination of Strength.

[34] (2019). Testing Hardened Concrete—Part 3: Compressive Strength of Test Specimens.

[35] Witkowska-Dobrev J., Szlachetka O., Malarski M., Czajkowska J., Miturski M., Nowak P., Dohojda M. (2023). Effect of Sewage on Compressive Strength and Geometric Texture of the Surface of Concrete Elements. Struct. Concr..

[36] Carabba L., Moricone R., Scarponi G.E., Tugnoli A., Bignozzi M.C. (2019). Alkali Activated Lightweight Mortars for Passive Fire Protection: A Preliminary Study. Constr. Build. Mater..

[37] Boggarapu V., Gujjala R., Ojha S., Acharya S., Venkateswara babu P., Chowdary S., kumar Gara D. (2021). State of the Art in Functionally Graded Materials. Compos. Struct..

[38] Benjeddou O., Ravindran G., Abdelzaher M.A. (2023). Thermal and Acoustic Features of Lightweight Concrete Based on Marble Wastes and Expanded Perlite Aggregate. Buildings.

[39] Grzeszczyk S., Janus G. (2021). Lightweight Reactive Powder Concrete Containing Expanded Perlite. Materials.

[40] Pramusanto P., Nurrochman A., Mamby H.E., Nugraha P. (2020). High Strength Lightweight Concrete with Expandable Perlite as the Aggregate. IOP Conf. Ser. Mater. Sci. Eng..

[41] Barbu S., Sabău A.-D., Manoli D.-M., Erbas R., Szlachetka O., Dzięcioł J., Baryła A., Dohojda M., Sas W. (2023). Analysis of the Water/Cement/Bentonite Ratio Used for Construction of Cut-Off Walls. Buildings.

[42] Dzięcioł J., Sas W. (2023). Perspective on the Application of Machine Learning Algorithms for Flow Parameter Estimation in Recycled Concrete Aggregate. Materials.

